# A review of evidence on mechanical properties of running specific prostheses and their relationship with running performance

**DOI:** 10.3389/fresc.2024.1402114

**Published:** 2024-06-19

**Authors:** Leila Rahnama, Kimberly Soulis, Mark D. Geil

**Affiliations:** ^1^ Rongxiang Xu College of Health and Human Services, California State University, Los Angeles, Los Angeles, CA, United States; ^2^ Employee Wellness Department, Wellstar Health System, Marietta, GA, United States; ^3^ Wellstar College of Health and Human Services, Kennesaw State University, Kennesaw, GA, United States

**Keywords:** prostheses, mechanical properties, stiffness, running, performance

## Abstract

**Background:**

Although mechanical properties of running specific prostheses (RSPs) can affect running performance, manufacturers do not consistently report them. This study aimed to review existing literature on RSP mechanical and structural properties and their relationship with running performance.

**Methods:**

A comprehensive search was conducted using keywords related to mechanical properties of RSPs and running performance. Search terms included stiffness and hysteresis, as well as performance outcomes including metabolic cost and running speed. Non-peer-reviewed and non-English publications were excluded.

**Results:**

Twenty articles were included in the review. Sixteen studies used a material testing machine to measure RSP material properties, and four articles used other techniques including 2D/3D video capture and force platforms. Both measurement techniques and reporting of outcomes were inconsistent, which limits the ability to draw broad conclusions. Additionally, several studies did not report the numerical data for material properties despite measuring them. Relatively few articles measured both material properties and running performance and assessed correlations.

**Conclusion:**

Several articles connected prosthesis properties to running performance. However, inconsistent measurement and reporting of mechanical properties, along with the multifactorial nature of the athlete-prosthesis system, limit the ability to draw broad conclusions regarding the relationship between material and structural properties and athlete performance. Current evidence may be useful for clinicians seeking ways to optimize RSP stiffness in a case-by-case basis; however, clinicians would benefit from more consistent and systematic comparisons of the attributes of different RSPs and their role in performance.

## 1 Introduction

Running specific prostheses (RSPs) have enabled many individuals with limb loss to improve their quality of life by engaging in more dynamic activities, including running. Awareness of the health benefits of exercise and the popularity of the sport, coupled with technological progressions in component materials and manufacturing, has encouraged more individuals with limb loss to use RSPs (1). One of the earliest efforts to enable more dynamic activities for users of prostheses was the Seattle Foot in the mid-1980's (2). Subsequent designs were specialized for running and sprinting (3). The majority of RSPs are made of carbon fiber, and they rely on storage of the energy generated from the athlete's body mass and acceleration and return of as much of that energy as possible to the body to contribute to the propulsive force needed for running (4–6). From a mechanical perspective, an RSP is a leaf spring that stores energy when deflected by the ground reaction force (GRF) during stance phase and returns energy during unloading (1, 5–7). Accordingly, the prosthesis shape and mechanical properties, such as damping and stiffness, affect the overall stiffness of the leg, and can therefore affect athlete performance (5, 7, 8). Therefore, material properties of RSPs could be considered a factor in the optimization of athlete performance.

As new application of materials emerged and new foot shapes enabled modification of energy-storage-and-return feet to the RSP concept, studies began identifying relevant outcome measures to quantify performance. For example, energy return efficiency was used to quantify how much of the absorbed energy was returned (modeled as an elastic spring) and not simply dissipated. An early study by Czerniecki et al. measured the efficiency of non-RSPs in running, finding very poor energy return efficiency in the traditional Solid Ankle Cushion Heel SACH foot (31%), better efficiency in the Seattle Foot (52%), and highest efficiency in a progenitor of modern RSPs, the Flex Foot (84%) (9).

Additional material properties and stiffnesses have been studied extensively in non-running-specific lower limb prostheses (10–20). Various studies have addressed properties of feet (18, 21), ankles (22, 23), and pylons (24, 25) in multiple planes, and many have correlated these properties to function in gait. However, because the dynamics of running are so different from walking (26, 27), including loading modes and magnitudes, deflection amounts, and loading frequencies and rates, it is difficult to translate findings about non-RSPs to understand RSP function.

The use of RSPs has not been without controversy. Researchers have addressed debates by officials and governing bodies whether athletes using RSPs gain biomechanical or physiological advantages over their able-bodied peers (28, 29). Indeed, the *Journal of Applied Physiology* published two articles in a “point/counterpoint” series titled, “Artificial limbs do make artificially fast running speeds possible” and “Artificial legs do not make artificially fast running speeds possible” (30, 31) For their part, some athletes believe prostheses limit their performance (6). Oscar Pistorius became a focus of debate when he sought to compete in Olympic events as well as Paralympic events, and himself once claimed that a competitor had been technologically advantaged through some modification to his RSP (32). The international prominence of the Olympics has brought significant attention to these debates and highlights the importance of understanding the function of RSPs and the link to athlete performance.

Therefore, the present study aimed to systematically review existing literature to characterize and compare numerical values of RSP material and structural properties and to evaluate how these properties affect athletic performance in individuals with limb loss.

## 2 Methods

### 2.1 The study guidelines

This study was conducted based on PRISMA guidelines (33) (Supplementary S1). The following research questions were formulated based on the aims of the study using the SPIDER tool (34) as follows: Sample: Users of Running-Specific Prostheses (RSPs), Phenomenon of Interest: Mechanical properties of RSPs, Design: Published literature, excluding gray literature, Evaluation: Effect of mechanical properties on running performance, Research Type: Quantitative or mixed methods, peer-reviewed studies excluding review articles.

### 2.2 Search methodology

We conducted a comprehensive search strategy through the following databases from inception until February 2024: PubMed, Web of Science, Scopus, Cochrane Library for Systematic Reviews, ScienceDirect, CINAHL, Ovid, EmCare via Ovid, ProQuest, ACM Digital Library, Compendex, and Google Scholar. Additionally, we searched the following publishers to ensure a thorough search: Elsevier, Nature, Frontiers, and Sage. Database-appropriate search syntax was used based on the following, intended to capture articles about both limb prostheses and their material properties:

(prosthe* OR cheetah OR blade) AND (run OR running) AND (mechanical OR stiff* OR energy OR hysteresis OR modulus) NOT (implant)

The reference lists of included articles and related systematic reviews were checked to find additional related studies, if any. Three authors (LR, KS, MG) searched all the databases and publisher platforms independently. They screened the titles, abstracts, and full-text articles to assess the eligibility. Any disagreement concerning the inclusion of a study was resolved by a single author (MG).

### 2.3 Identification and selection of the studies

We included all studies in which at least one RSP was assessed for mechanical properties and studies that evaluated the relationships between these mechanical characteristics with any aspects of the running performance. The gray literature including conference papers, theses, repository data, or any non-peer-reviewed publications was not included. Only articles written in English were included.

## 3 Results

### 3.1 Overview of evidence

**Figure 1:**
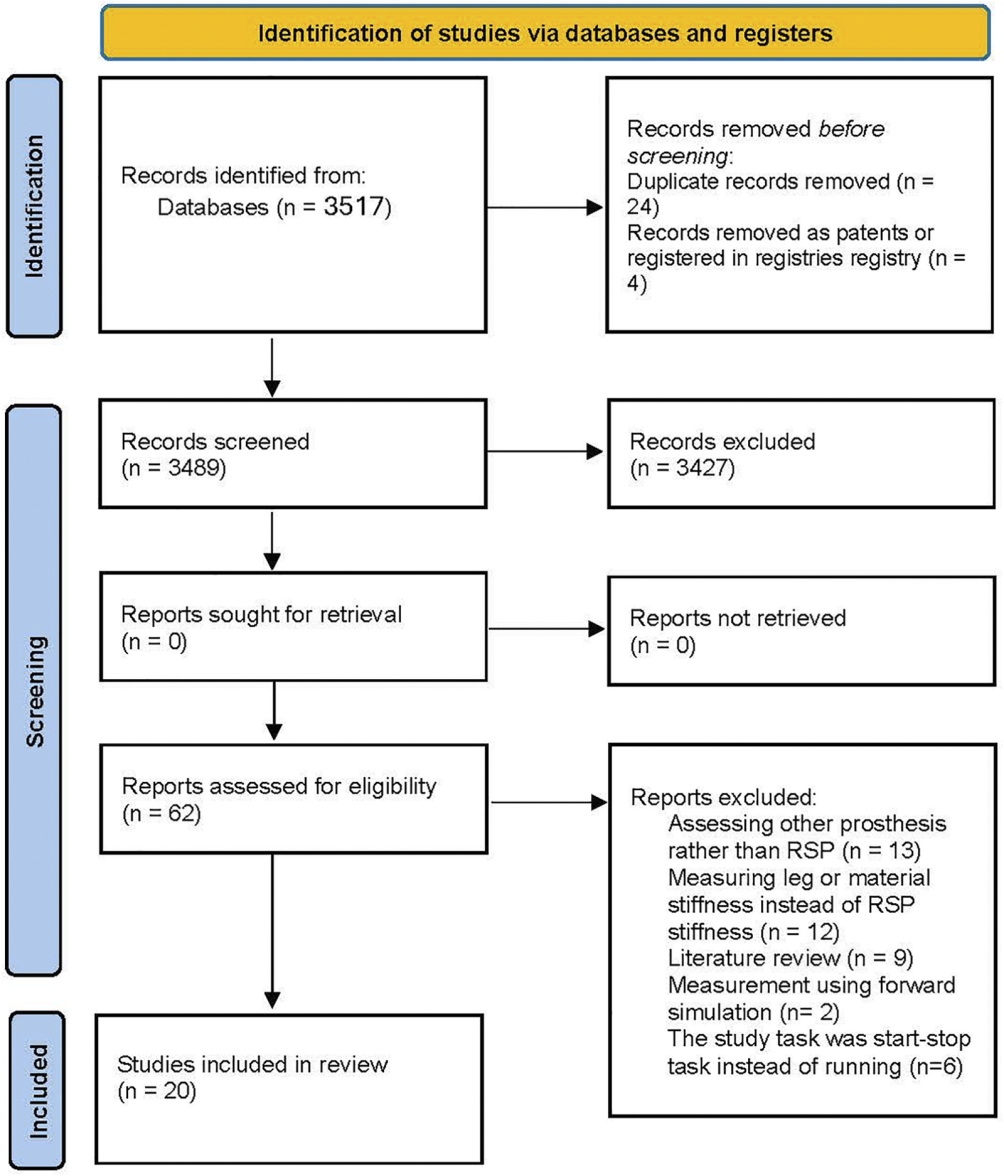
Number of articles identified, excluded, and reviewed.

The search process generated 3,517 articles containing papers from searched databases and citation references in included articles. Figure 1 shows the total number of articles screened and the number of articles excluded for different reasons. Although our search syntax included “NOT implant” to avoid studies on joint or dental implants, we still had some in search results, which were excluded later. Six studies were from the same research group and sometimes used the same data set, but we reported them as separate studies. The total number of included articles was twenty.

### 3.2 Study characteristics

We included studies in which the RSP stiffness or hysteresis (by any given name) were measured through two fundamental techniques: direct loading/unloading or mathematical calculation using kinetic data, and all articles that had assessed the relationship between RSP stiffness (manufacturer classified category, or the exact value) or hysteresis with any aspects of running performance including running speed, step frequency, energy cost, or metabolic cost in amputees.

### 3.3 Mechanical properties measurements

The numerical values of RSP stiffness were measured/categorized in 20 studies. In 10 studies, a material testing machine was used to measure RSP stiffness (Table 1). Six studies used previously established data and a force-sensing treadmill to measure stiffness (Table 1). The other four studies used either a force-sensing treadmill or a constant weight to measure stiffness (1, 5, 46, 47). Mechanical stiffness was calculated as load divided by deflection. Although 20 studies measured/categorized the mechanical properties of RSPs, only 10 studies reported numerical values for prosthetic stiffness, while 6 studies reported the load-deflection graph (4, 8, 32, 36, 38, 47), from which stiffness could be secondarily interpreted.

**Table 1:** Studies using material testing machine to measure stiffness/hysteresis.

Author (year)	Running specific prostheses (RSP)	Loading rate (LR)/peak load (PL)	Foot orientation	Numerical value stiffness (N/mm)	Numerical value hysteresis	Testing machine
Brüggemann Gert-Peter (35)	Cheetah, Össur, Iceland	LR = 1,000 mm/min PL = 1,500 N	Vertical (loading unloading)	Left keel = 38.7 Right keel = 38.9	Left keel = 4.9% Right keel = 5%	Material testing machine T1-FR020TN.A50 (Zwick GmbH & Co, Ulm, Germany)
Dyer et al. (32)	Elite Blade, Chas A Blatchford & Sons Ltd, Basingstoke, UK	LR = 50 mm/min PL = 2,000 N	Vertically placed in two conditions: Fixed at the prostheses distal end (FDE)^a^. Partial slide then fixed (PSF)^a^.	FDE^a^ = 69 PSF^a^ = 76	Not reported	Testometric Company Ltd., Lancashire, UK
Dyer et al. (4)	Two Elite Blade (specifications unknown), Chas A Blatchford & Sons Ltd, Basingstoke, UK	LR = 50 mm/min PL = 2,000 N Stiffness is given at 1,950) UDE^a^ 1,500 N PL = 3,500 N	Vertically placed three conditions: Fixed at the prostheses distal end (FDE)^a^. Partial slide then fixed (PSF)^a^ and Unfixed distal end (UDE)^a^.	RSP 1: FDE^a^ = 60 PSF^a^ = 58 UDE^a^ = 34 RSP2: FDE^a^ = 48 PSF^a^ = 42 FDE^a^ = 53	Not reported	Testometric Company Ltd., Lancashire, UK
Hawkins et al. (8)	Össur “Flex Run” Cat6Hi prosthetic running foot.	PL: Condition1 = 1.2 Condition2 = 2.81 Condition3 = 2.83 Condition4 = 1.71	4 conditions with different friction with ground: low, medium, and high friction, and loading on a mobile RSP.	Not reported	Not reported	Instron 8,872 hydraulic test machine Series of sine-wave oscillations (Frequency = 0.5 Hz)
Hamzah and Hameed Mirza (36)	Custom manufactured RSP: Two designs (shapes)	RL = not reported PL = 1,600 N	0 and 25 degrees of ankle dorsiflexion	Not reported	Not reported	A material testing machine, no data on the manufacturer.
Abbod and Faidh-Allah (37)	Custom manufactured: Glass fiber (GF) vs. carbon fiber (CF) RSP	RL = not reported PL = 1,500 N	Vertically Compressed.	GF = 28 CF = 57	Not reported	A material testing machine, no data on the manufacturer.
Beck et al. (38)	55 RSPs, different stiffness categories and shapes (C vs. J): Össur, Reykjavik, Iceland Freedom Innovations, Irvine, CA, USA), and Ottobock, Duderstadt, Germany.	LR = 100 N/s PL = 2,724 N	Three loading and unloading cycles in six different mounting angles: 0°, α3 = 15.0°, α6 = 10.0°, β3 = 20.0°, and β6 = 25.0°.	Estimated range from 17.7 (1E90 cat. 1, 25°) to 61.1 (Catapult cat. 7, no sole, neutral angle)^a^	Ranged from 3.7% to 8.1%	Materials testing machine (Instron Series 5,859, Norwood, MA, USA)
Beck et al. (39)	Freedom Innovations Catapult FX6, Irvine, CA; Össur Flex-Run, Reykjavik, Iceland; Ottobock 1E90 Sprinter, Duderstadt, Germany	Using data from Beck et al. study and ground reaction force (GRF) from a running task.	Assessment has been done by running on a treadmill in different running speed from 3 to 7 m/s.	25.4 + 3.0, 26.1 + 3.4, 27.1 + 4.0, 28.0 + 4.8 and 28.6 + 5.6	Not reported	Using data from Beck et al. study and a 3-dimensional force-measuring treadmill (Treadmetrix, Park City, UT)
Beck et al. (40)	Freedom Innovations Catapult FX6, Irvine, CA, and Össur Flex-Run, Reykjavik, Iceland; and Ottobock 1E90 Sprinter, Duderstadt, Germany)	Using data from Beck et al. study and GRF from a running task	Assessment has been done by running on a treadmill in different running speed from 3 to 2.5 m/s.	Not reported	Not reported	Using data from Beck et al. study and a 3-dimensional force-measuring treadmill (Treadmetrix, Park City, UT)
Beck et al. (41)	Freedom Innovations Catapult FX6, Irvine, CA, USA; Össur Cheetah Xtend, Reykjavik, Iceland, Ottobock 1E90 Sprinter, Duderstadt, Germany.	Using data from Beck et al. study and GRF taken from a running task	Assessment has been done by running on a treadmill in different running speed from 3 to 2.5 m/s.	Ranged from 19.3 to 29.6	Not reported	Using data from Beck et al. study and a 3-dimensional force-measuring treadmill (Treadmetrix, Park City, UT)
Taboga et al. (7)	Freedom Innovations Catapult FX6, Irvine, CA, Ottobock 1E90 Sprinter Duderstadt, Germany, and Össur Cheetah Xtend, Reykjavik, Iceland).	Using data from Beck et al. study and GRF from a running task	Assessment has been done by running on a treadmill in different running speed from 3 m/s and incremented by 1 m/s.	Not reported	Not reported	Using data from Beck et al. study and a 3-dimensional force-measuring treadmill (Treadmetrix, Park City, UT) and 3D motion capture system (Vicon Nexus, Oxford, UK)
Taboga et al. (42)	Freedom Innovations Catapult FX6, Irvine, CA, Ottobock 1E90 Sprinter Duderstadt, Germany, and Össur Cheetah Xtend, Reykjavik, Iceland).	Using data from Beck et al. study and GRF from a running task	Using data from Beck et al. study Assessment has been done by running on a treadmill in different running speed from 3 m/s and incremented by 1 m/s.	Not reported	Not reported	Using data from Beck et al. study and a 3-dimensional force-measuring treadmill (Treadmetrix, Park City, UT) and 3D motion capture system (Vicon Nexus, Oxford, UK)
Guzelbulut et al. (43)	E91 Runner Ottobock, Duderstadt, Germany	No information on loading force	A fixture was used to limit the horizontal motion of the RSP/No information on mounting position	Not reported	Not reported	A material testing machine, no data on the manufacturer.
Tacca et al. (44)	Freedom Innovations Catapult FX6, Irvine, CA, USA; Össur Cheetah Xtend, Reykjavik, Iceland, Ottobock 1E90 Sprinter, Duderstadt, Germany.	Using data from Beck et al. study and GRF from a running task	The study involved further analysis of the data collected by Beck et al.	Not reported	Not reported	Further analysis of the data collected by Beck et al.
Doyen et al. (45)	Ottobock 1E90 Sprinter	Three rows of 50 mm loading- 50 mm unloading at each position with the loading rate of 1 mms^−1^	Several load line from −40 to +40 mm at different prosthesis-ground angles of 0–30 degrees.	Maximum stiffness for the loading alignment and prosthesis ground angle was reported as 28.9 and 27.1 kNm^−1^, respectively.	The maximum dissipation percentage in various conditions: 15.1%, 19.9%, 13.5%, 14.6%, 18.7%, 9.7%	An electromechanical testing machine (Instron 5,967) with a 30 kN loadcell (2,580 series static; class0.5)
Shepherd	Three carbon fiber fabricated feet (three shapes)	Loads were applied vertically with 200 N increments up to 2,000 N.	Purely vertical load	Not reported	Not reported	Universal material testing system (MTS, USA)

^a^ RSP, running specific prostheses; LR, loading rate; PL, peak load; FDE, fixed at the prostheses distal end; PSF, partial slide then fixed; UDE, unfixed distal end; GF, glass fiber; CF, carbon fiber; GRF, ground reaction force.

In one instance (38), stiffness values were not reported, but quadratic functions were provided for the nonlinear load-deformation characteristics of a variety of RSPs. To estimate stiffness values, we used a technique similar to the same research group in another paper (39). Using a force value of 2 KN [based on loading graphs in (38)], we determined the corresponding deformation from the quadratic equations, and then divided 2 KN by the associated deformation.

Hysteresis and efficiency are related concepts, and there is some inconsistency in reporting. Three studies reported values for hysteresis (35, 38, 45) and two studies reported energy efficiency (8, 36). Hawkins et al. defined efficiency as the compression phase energy divided by the “rebound phase” energy, expressed as a percentage (8). While hysteresis is often calculated as the difference between the so-called compression phase energy and the rebound phase energy, Beck et al. divided this difference by the compression phase energy and expressed hysteresis as a percentage (38). Doyen et al. calculated hysteresis using a damping parameter expressed as the ratio between the area of the loop formed by loading and unloading curves and the area beneath a linear curve (45).

### 3.4 The association of mechanical properties and performance

Eight studies assessed the relationship between the mechanical properties of RSPs and running performance indices, including running speed and cost of transport (CoT) (7, 39–44, 48). Stride or step frequency, GRF, and contact time were assessed in some of these studies. Since these indices are more general measures of kinematics or kinetics than performance, we have not reported them in the current review. We used running speed and cost of transport as pivotal indicators of running performance due to their frequent usage in assessing running efficiency and overall performance (49, 50). These metrics are commonly quantified through various indices such as time and VO_2_ Max, respectfully (49, 50). Any study that only evaluated the relationships between other characteristics of RSPs (not mechanical properties), including shape or model, was excluded.

The association between running speed and RSP stiffness was assessed in six studies (7, 39, 42–44, 48). However, three studies (7, 39, 44) were based on the same data set. They measured the effect of prosthesis stiffness (using three manufacturer-defined RSP stiffness categories) on running outcomes such as temporal and spatial parameters, GRF, and leg stiffness. These studies concluded that effects of prosthetic stiffness diminished at faster running speeds, suggesting stiffness might have more of an influence on distance running performance vs. sprinting (39) These authors found that that leg stiffness—the overall quotient of peak vertical ground reaction force and peak leg spring compression—has differential sensitivity to prosthetic stiffness and running speed in athletes with bilateral transtibial amputation. Running speed was significantly inversely associated with leg stiffness, which incorporates both RSP stiffness and residual limb biomechanics and physiology. Toboga et al. (42) reported a similar finding in unilateral transtibial amputees using the same methods and same RSPs as their study in individuals with bilateral transtibial amputation. Nevertheless, in their recent publication, the same research team revealed that the step frequency rose in correlation with higher speeds, and this effect was mitigated when participants used a stiffer RSP compared to a less stiff one (44).

Metabolic cost of running or CoT was measured in two studies (40, 41). One study was conducted on individuals with unilateral transtibial amputation that showed no association between RSP stiffness and CoT (40). The other study was done on individuals with bilateral transtibial limb loss and revealed a positive association between these two factors with lower RSP stiffness reducing CoT (41).

Only one study reported the relationship between RSP hysteresis and running speed (7). This study on bilateral transtibial amputees showed a negative correlation between RSP hysteresis and maximum running speed.

## 4 Discussion

The objective of this study was to explore how RSP mechanical properties affect performance.

This review showed that several factors, including the method of mounting the prosthesis to the test machine, friction abatement, and the humidity and temperature of the surrounding environment, can affect the comparability of the testing results between studies (8). Loading rate is another influencing factor for which there is no related standard guideline in the literature for running. There was a wide variation in loading rate between the studies ranging from 50 (4, 32) to 1,000 mm/min (35), with some studies limited by equipment and others seeking to replicate different loading rates observed in running. Because no material is perfectly elastic, and because properties of viscoelastic materials depend on loading rate, a given prosthesis might display different properties when loaded more rapidly or slowly, emphasizing the need for standardization, or at least careful reporting. These discrepancies between techniques make it difficult to compare the findings of different studies and might at least partially explain discrepancies in reported load-deflection functions in the included studies. Some studies reported a linear load-deflection function (35), while others reported a polynomial profile (38, 47). These differences could reflect properties of different feet, or variability in loading methods. The applied peak load also varied among the studies. Some studies loaded feet to a maximum of 1,500 N (4, 37), while some went up to 3,500 N (4). Most manufacturers do not report standardized technical specifications for their prostheses, making objective distinction challenging (10). Providing a standard guideline for reporting such laboratory measurements would enhance the quality and consistency of material measurement tests, ultimately improving our understanding of the efficacy of running prostheses.

Beck et al. (38) reiterated this point by attempting to duplicate the methods used by Brüggemann et al. (35) to assess the stiffness of a particular RSP, including applied force, neutral foot orientation angle, displacement-controlled loading mode, and loading rate. This method produced a stiffness of 34.2 kN/m. Then, they tested the same foot with a higher applied force, force-controlled loading mode, and plantarflexed orientation angle. This resulted in a stiffness for the same foot of 29.2 kN/m. This experiment, along with other differences found in this review, demonstrates that testing of prostheses should be considered assessment of “structural” properties, not “material” properties. Testing of the properties of a single material should show consistency across loading magnitude (within the elastic region). Loading orientation might vary due to material isotropy, and rate-dependence can reveal a viscoelastic element to the material. With RSPs, a test is considering multiple materials, and can be dependent on load magnitude, rate, and direction. Consequently, tests are addressing the entire structure and should not be inferred as being exclusively descriptive of, say, the material properties of the carbon fiber. Furthermore, although RSPs appear to be largely elastic materials, there is a lack of data on their viscoelastic properties, given the relatively low number of studies reporting hysteresis, and a lack of testing in conditions that duplicate actual use, including foot/ground interfaces such as soles. Shepherd et al. (47) originally aimed to incorporate soles into their model but deferred this aspect into future research. They cited the complexity of modeling soft viscoelastic soles as a challenging factor. However, Doyen et al. (45) conducted a comparison of the effects of two sole types (running and spike) on blade stiffness. They reported a linear evolution of the instantaneous stiffness beyond a specific displacement threshold, occurring due to lack of the full contact between the sole and the flooring.

Even within the construct of material vs. structural properties, prosthetic foot stiffness and shape are separate but related considerations. RSP models are generally C-shaped or J-shaped, both affording a long cantilever beam over which elastic deformation (based on material stiffness) is distributed. The shape affects mounting and height (42), and likely affects the natural frequency of the prosthesis. Shorter cantilever beam lengths may necessitate increased stiffness in order to prevent material failure at areas of stress concentration.

Inferences about material properties have varied as well. Several studies, including many within the same author groups, have found specific RSP properties such as stiffness to be relevant to certain running outcomes but irrelevant to others (7, 38, 40, 51). Some researchers have concluded that the stiffness or shape of the prosthesis could alleviate the kinematic asymmetries in athletes with unilateral amputation (40). These diverse conclusions indicate the complex role the prosthesis plays in running biomechanics and performance and suggest that the relationship between RSP properties and running performance cannot be characterized with a single conclusive result.

A total of five studies assessed the association between RSP stiffness and running speed. Three studies concluded that RSP stiffness is not the primary factor affecting running speed in high-speed sprints (7, 39, 42). However, the strength of the conclusion is limited because all three studies were conducted by the same research group and primarily used the same data. It is noteworthy to mention that a recent study by Barnett et al. found that running speed was independent of the RSP stiffness (48). In contrast to these findings, Guzelbulut et al. reported that an increase in blade stiffness, coupled by enhancement in shape, resulted in improved sprint performance, measured as horizontal velocity (43). However, they considered both factors, stiffness and shape, together. More studies are needed to help improve our understanding of the effects of RSP stiffness on running speed.

The metabolic cost of running or CoT was assessed in two studies by the same research team, one focused on individuals with unilateral limb loss (40) and the other on bilateral limb loss (41). Both articles used previously collected data on RSP material properties to make inferences on athlete performance. While the authors concluded that prosthetic stiffness affects CoT in individuals with bilateral limb loss, the result in unilateral limb loss was not significant. These different results highlight the importance of understanding the role of the residual limb musculature and the contralateral limb (when present) in dynamically adjusting overall leg stiffness to optimize gait and potentially mitigate the effects of suboptimal prosthesis properties.

To compound inconsistencies in measurement techniques, the literature also reveals inconsistency in reporting. For instance, some articles were limited to reporting descriptive data and graphs, lacking the exact value of variables. Access to full numerical data sets can lead to subsequent research and insight into the efficacy of each prosthesis for a particular activity or more personalized prescription of prostheses.

The review focused on stiffness as the most commonly reported mechanical property, with relatively fewer studies addressing hysteresis. Evidence is lacking concerning the effect of other properties, such as natural frequency and vertical displacement. A recent study found that the forward distance covered by the participant's center of mass (COM) during the contact period is a pivotal factor influencing peak running speed (52). Another highlighted the impact of stiffness on leg length specifically during midstance, making it important for athletes with unilateral amputation (53).

Finally, it is noteworthy that all the studies in the review were focused on prostheses for adults. Given that RSPs have been designed for children, future research should include pediatric RSPs and appropriate loading specifications to mimic running in children.

In general, manufacturers do not report actual structural properties of prosthetic feet. Therefore, clinicians who wish to make objective comparisons about RSP properties rely on the literature. However, testing methods and reporting in research are inconsistent. For these reasons, the prescription of RSPs is usually subjective and based on either provider experience or athlete comfort and not based on scientifically-based guidelines (5).

### 4.1 Limitations

Our findings in the current review are limited to articles written in English. There might be some studies in which material properties of RSPs have been assessed, but the reports are not in English. We also saw some conference publications and thesis projects on assessing material properties of RSPs or evaluating their relationships with running performance. However, because they did not undergo a standard journal peer-review process, we did not include them in this study.

### 4.2 Conclusions

The current review showed inconsistency in assessment and reporting of mechanical properties of RSPs, and a small number of properties commonly tested. Consequently, not all possible associations of material properties and athletic performance have been assessed in amputees.

Studies on the relationship between RSP mechanical properties and athlete performance illustrate the multifactorial nature of this research, which must consider the prosthesis as a part of a complex kinematic and dynamic system in concert with the athlete. Definitive conclusions are therefore difficult to obtain and could benefit from more complex modeling.
